# Six-year clinical outcomes of implant-supported acrylic vs. ceramic superstructures according to the All-on-4 treatment concept for the rehabilitation of the edentulous maxilla

**DOI:** 10.1007/s10266-021-00605-4

**Published:** 2021-04-10

**Authors:** Mustafa Ayna, Fatih Karayürek, Søren Jepsen, Marie Emmert, Yahya Acil, Jörg Wiltfang, Aydin Gülses

**Affiliations:** 1 Department of Periodontology, University Hospital Bonn, Bonn University, Bonn, Germany; 2 Department of Periodontology, Cankiri Karatekin University, Cankiri, Turkey; 3 Department of Oral and Maxillofacial Surgery, Universitätsklinikum Schleswig Holstein, Christian Albrechts University, Campus Kiel, Arnold-Heller-Straße 3, 24105 Kiel, Germany

**Keywords:** Edentulous, Maxilla, Acrylic, Ceramic, Bone loss, Immediate loading

## Abstract

The aim of the current study was to document the long-term clinical results of the use of two prosthetic techniques for the rehabilitation of completely edentulous maxillae according to the “All-on-Four” concept: Fixed, screw-retained prosthesis mounted on a chrome-molybdenum framework with (1) metal-ceramic veneers and (2) Acrylic prosthesis with acrylic resin prosthetic teeth. A total of 34 patients were assigned to subgroups according to their own preference of the superstructure type (ceramics [*n*: 17] or acrylic resin [*n*: 17]). Prosthetic complications, marginal bone loss, plaque accumulation, bleeding on probing, bite force and oral-health-related quality of life were assessed over a period of 6 years. Marginal bone loss around implants of the ceramic group remained well within the limits for ‘success’, as defined by the 2007 Pisa consensus over the time (1.43 ± 0.35 mm). However, marginal bone loss was significantly more pronounced around the implants in the acrylic group (2.15 ± 0.30) and the difference between two groups was statistically significant (*p*: 0.00). Bleeding on probing and plaque accumulation showed also positive correlation with marginal bone loss. Both acrylic and ceramic suprastructures appeared to be equivalent after 6 years; however, ceramic suprastructures revealed superior clinical results in terms of bone loss and plaque accumulation. Current study determines the long-term clinical outcomes of different prosthetic management alternatives in All-on-Four and aids to increase dental professionals’ ability to meet the patients’ expectations.

## Introduction

Due to the patients’ demands regarding re-establishment of function, phonation and esthetics within the shortest possible time, immediate loading concepts are increasingly becoming the preferred treatment option in the daily dental practice [1]. Since 2003, full fixed arch prosthesis and immediate function via the so-called “All-on-4” concept outshines as a fast and reliable therapy option, which was first intended for the rehabilitation of the edentulous mandibles [2]. Two years after the description of the technique for the rehabilitation of the mandibles, Maló et al. have also demonstrated high survival rates for immediate functional loading of four implants as a support for a full-arch maxillary prosthesis [3].

“All-on-Four” bases on the load-bearing capacity of the jaws [4] and allows basically for two different types of superstructures [5, 6] regarding the final prosthetic protocol; metal–ceramic implant-supported fixed prosthesis with ceramic veneers and implant-supported fixed acrylic resin prosthesis with a metal framework and acrylic resin prosthetic teeth.

In the “All-on-4” concept, patients’ preferences and financial status may be decisive factors in the selection of the final prosthesis as there is mostly a considerable difference in the price for different types of superstructures (laboratory costs are approximately 2000 USD or acrylic and 5500 USD for ceramics, respectively) [6]. Therefore, determining the long-term results of different prosthetic management alternatives would help dental practitioner to gain insight of the cost–benefits and improve the prediction of clinical outcomes.

In the literature, there are comprehensive studies focusing on the clinical parameters and survival rates of the implants placed and loaded according to the “All-on-Four” protocol [7, 8].

However, effects of different superstructures on the clinical outcomes were mostly overlooked. Considering the existing literature on the comparative assessment of acrylic vs. ceramic superstructures [6], it could be particularly hypothesized that implants supporting acrylic superstructures could exhibit higher inflammatory changes and concomitant marginal bone resorption than those loaded with metal-ceramic prosthesis. To the best of our knowledge, long-term results for acrylic superstructures with metal frameworks mounted on four implants according to the “All-on-Four” technique in the edentulous maxilla have not been investigated thus far. Therefore, the aim of the current study was to document the long-term clinical results of the use of two techniques for the rehabilitation of completely edentulous maxillae according to the “All-on-Four” concept: Fixed, screw-retained prosthesis mounted on a chrome-molybdenum framework with metal-ceramic vs fixed, screw-retained prosthesis mounted on a chrome-molybdenum framework with acrylic prosthesis with acrylic resin prosthetic teeth.

## Materials and methods

### Study design

Data of the patients who were treated between May 2013 and January 2014 for complete rehabilitation of the edentulous maxilla with immediately loaded implant-based fixed dentures according to All-on-Four protocol were screened for participation retrospectively. The study was approved by the Ethics Review Committee of MMAG (NEAH/12.15.2015#498). Patients were assigned to subgroups according to their own preference of the superstructure type (ceramics or acrylic resin).

### Inclusion criteria

- Rehabilitation of the edentulous maxilla with ‘All-on-4’ concept;
- natural dentition or tooth/implant supported fixed dentures of the mandible;
- absence of medical contraindications for oral surgical procedures (ASA I/II);
- regular attendance of dental recall appointments at regular intervals of 6 months;
- ability to maintain personal oral hygiene.

### Exclusion criteria

- Patients with uncontrolled systemic diseases and/or having possible contraindications for implant surgery which could jeopardize the osseointegration (uncontrolled diabetes, osteoporosis, psychiatric disorders, pregnancy, etc.;
- medication negatively affecting the osseointegration process (bisphosphonates, corticosteroids, serotonin re-uptake inhibitors, etc.);
- active inflammatory processes or neighboring pathologies at the implant recipient site;
- radiotherapy to the head and neck region in the last 24 months;
- bruxism;
- requirement of bone augmentation during implant placement;
- poor oral hygiene and/or compliance;
- nicotine/alcohol/drug abuse.

### Surgical procedure

Standard dental impressions were taken as if the patient were to receive an immediate maxillary denture after extractions. A finished complete maxillary denture was fabricated by dental laboratory for immediately postoperative modification as a provisional fixed restoration. Occlusal registration had been conducted. Preoperative pictures including the smile line, upper lip raised and in resting position were recorded. The vertical dimensions were ensured.

**Fig. 1:**
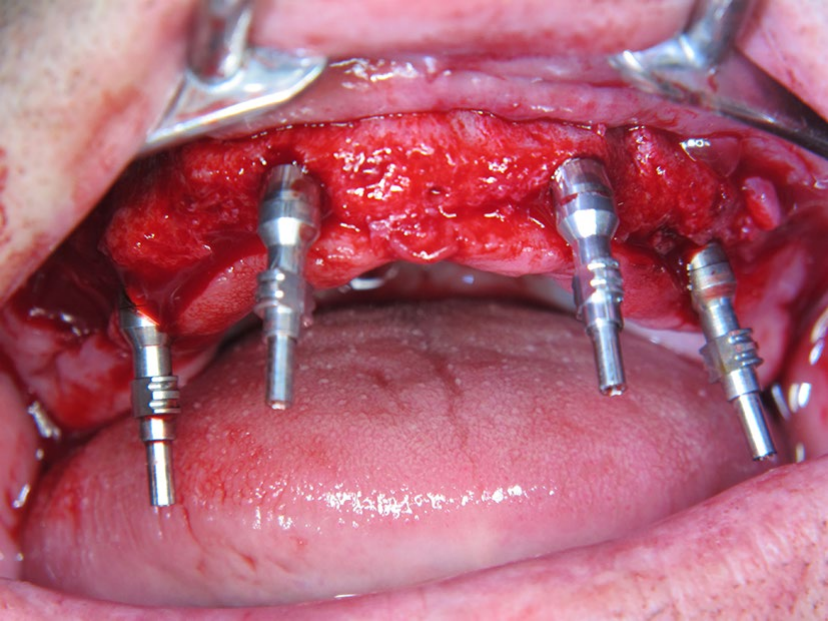
Placement of the impression copings prior to primary closure

All procedures were performed under local anesthesia (Articaine-hydrochlorid [72 mg/1.8 ml] with epinephrine [0.018 mg/1.8 ml] 1:100,000). All of the patients received four implants (Nobel Biocare™, Göteborg, Sweden) according to the “All-on-Four” protocol and the manufacturer’s guidelines in terms of angulation of the distal implants. Implant sizes were 13 mm mesial and 16 mm distal, the longer distal implants providing bi-cortical anchorage via the alveolar crest and the cortical bone of the sinus floor in the edentulous maxillae: a mucoperiosteal flap was raised at the ridge crest with relieving incisions on the buccal aspect in the molar area. When a high smile line or irregular/thin bone crest were present, bone reduction via an ostectomy was performed. The exact position of the anterior sinus wall was identified via a small (4 mm) bony window created with a round bur. The implants and abutments were placed in one position at a time, starting with the posterior implants. A special guide (Edentulous guide, Nobel Biocare™, Göteborg, Sweden) was used to assist implant and abutment placement to ensure the centrally placement of the implants according to opposing dentition and at the same time to find the most appropriate position and inclination for optimal implant anchorage and prosthetic support. The posterior implants were inserted with an angulation of 30–45°. The insertion of the implants followed standard procedures, except that, under preparation and bi-cortical anchorage—if possible—was used to achieve a peak insertion torque of at least 35 N/cm. Prior to primary closure and suturing of the flap with 3–0 non-resorbable sutures, impression copings were placed (Fig. 1).

All implant sizes were 4 × 13 mm for mesial and 4 × 16 mm for the distal implants. All implants were immediately loaded within 24 h. Antibiotics (Amoxicillin 875 mg/clavulanic acid 125 mg) were given 1 h prior to surgery and two times a day for 6 days thereafter. Anti-inflammatory medication (Ibuprofen, 600 mg × 4/day) was prescribed for 5 days.

### Immediate prosthetic procedure

**Fig. 2:**
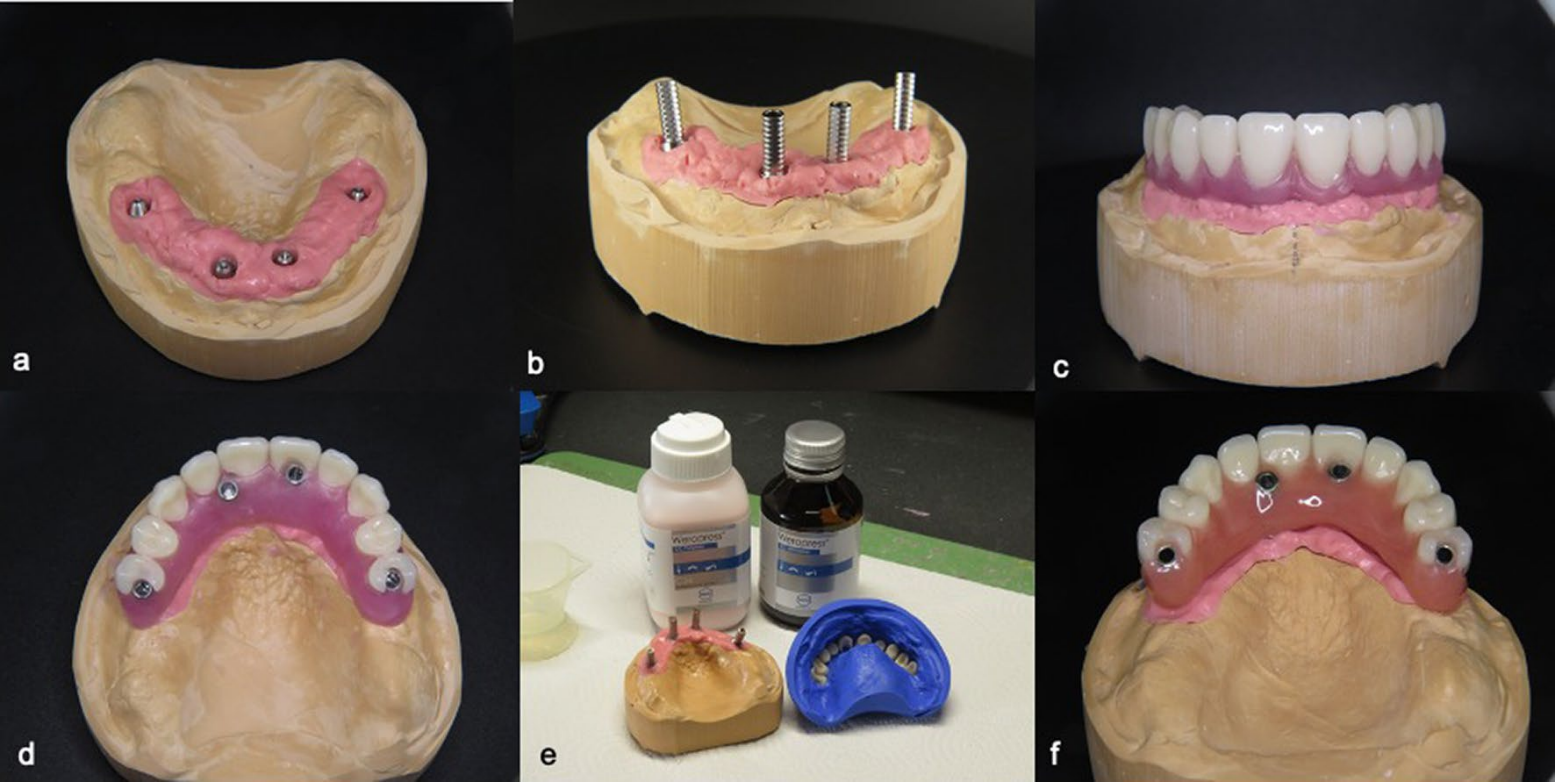
Production of the immediate acrylic prosthesis. **a** Completed master cast. **b** Placement of the titanium temporary abutments. **c** and **d** Acrylic teeth in wax set-up. **e** Preparation of high-density acrylic prosthesis. **f** Immediate acrylic prosthesis mounted on cast model

**Fig. 3:**
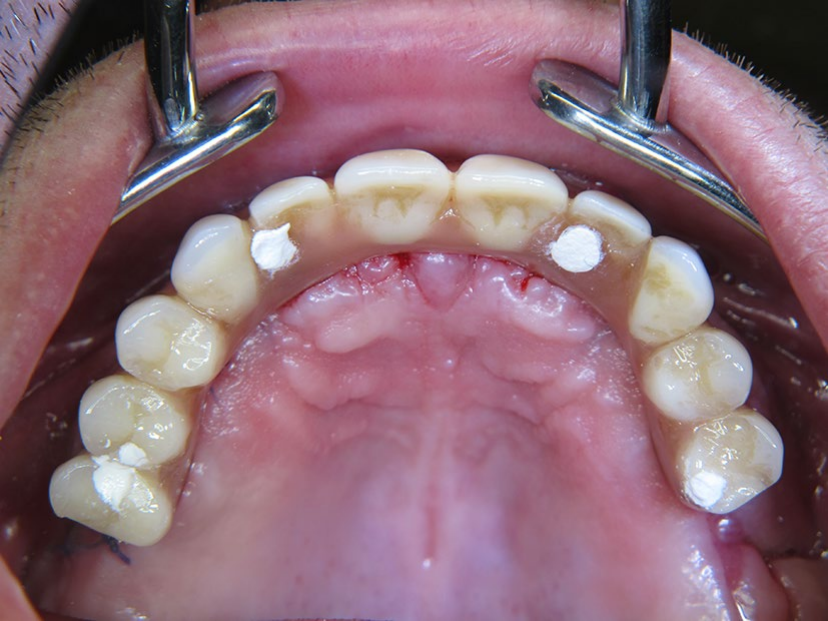
Adjustment of the immediate acrylic prosthesis in situ. Please note that cantilevers have been avoided

In both groups, prior to implant placement, an acrylic maxillary denture was fabricated for modification as a provisional fixed restoration. Following implant insertion, impression copings were placed on the implants. The positions of the impression copings were marked, and drill holes were made into the plastic impression tray. The impression material has been applied and an open tray impression has been taken. At the laboratory, implant replica multi-unit abutments were connected to impression copings. Afterwards, a preliminary model is poured and after sufficient time for stone to set, screws were loosened, and impression tray has been removed. The impression copings were taken from the impression material and titanium temporary abutments were placed on the implant replicas in the preliminary model. A high-density screw-retained implant-supported acrylic resin prosthesis was manufactured at the dental laboratory and mounted within 24 h post-surgery. (Figs. 2a–f and 3) All centric and lateral contacts were controlled with articulating paper at 40 µ and adjusted to obtain an optimal occlusal contact.

### Final prosthetic procedure

**Fig. 4:**
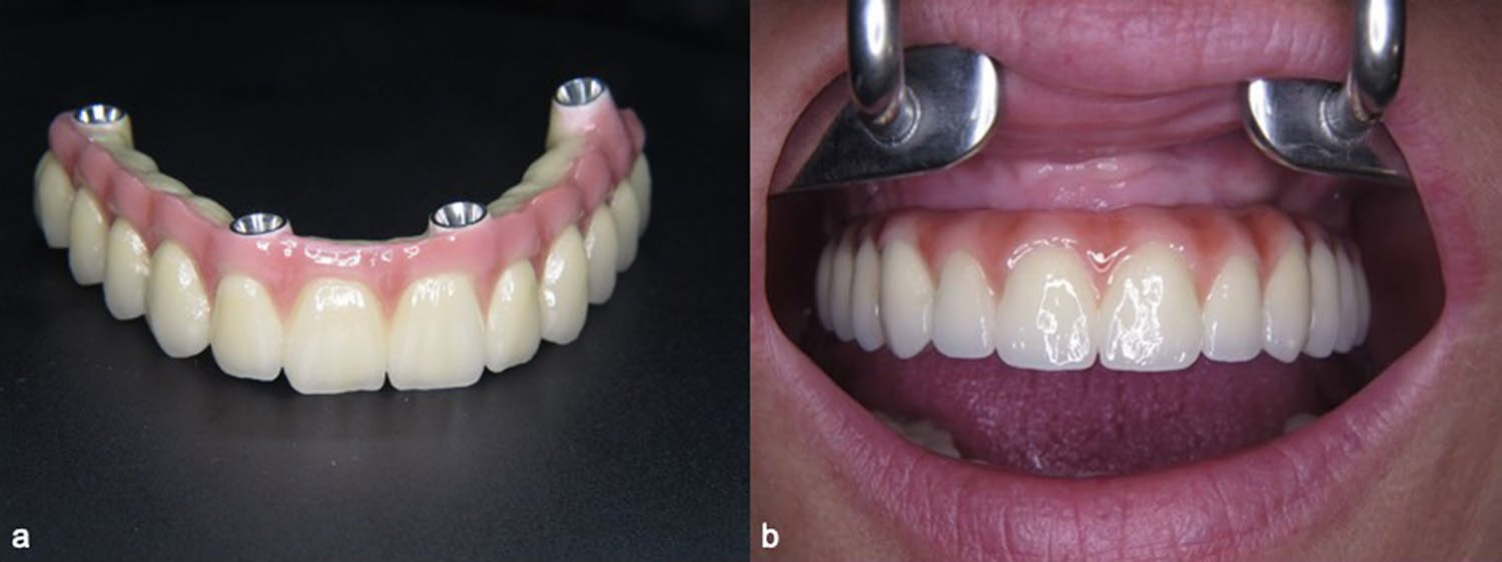
**a** Chrome–molybdenum framework-based metal–ceramic prosthesis **b** Adjustment in situ

**Fig. 5:**
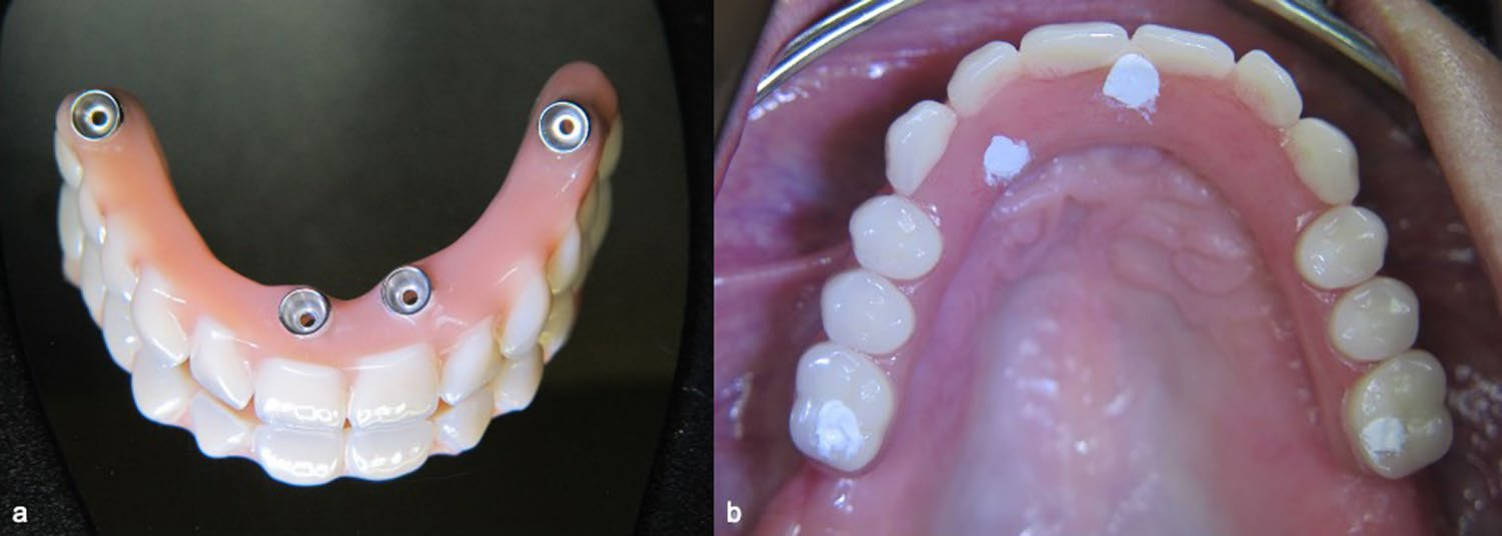
**a** Acrylic resin prosthesis with metal framework and acrylic resin prosthetic teeth **b** Adjustment in situ

All final prostheses were delivered 3 months after surgery. For the patients, who have preferred to receive an acrylic prosthesis, a chrome–molybdenum framework with a quadrilateral shape and two distal extensions was fabricated using CAD/CAM technology, and an acrylic resin implant-supported prosthesis with acrylic resin prosthetic teeth was prepared (Fig. 4) The patients who have decided to receive ceramic superstructures, a chrome–molybdenum framework was fabricated using CAD/CAM technology and a metal-ceramic implant-supported fixed prosthesis was connected to the implants (Fig. 5). In both groups, cantilever length was determined according to 1.5–2xA-P-spread rule as previously described by Malo et al. [3], which allow a 10–12 mm posterior cantilever extended to the first molar regions.

### Outcome parameters

All parameters including implant survival, prosthetic complications, marginal bone loss, probing pocket depth (PPD) and bleeding on probing (BOP), plaque accumulation, measurement of occlusal forces, and oral-heath-related quality of life were documented for each implant immediately after implant insertion and with 1-year intervals during a period of 6 years.

**Fig. 6:**
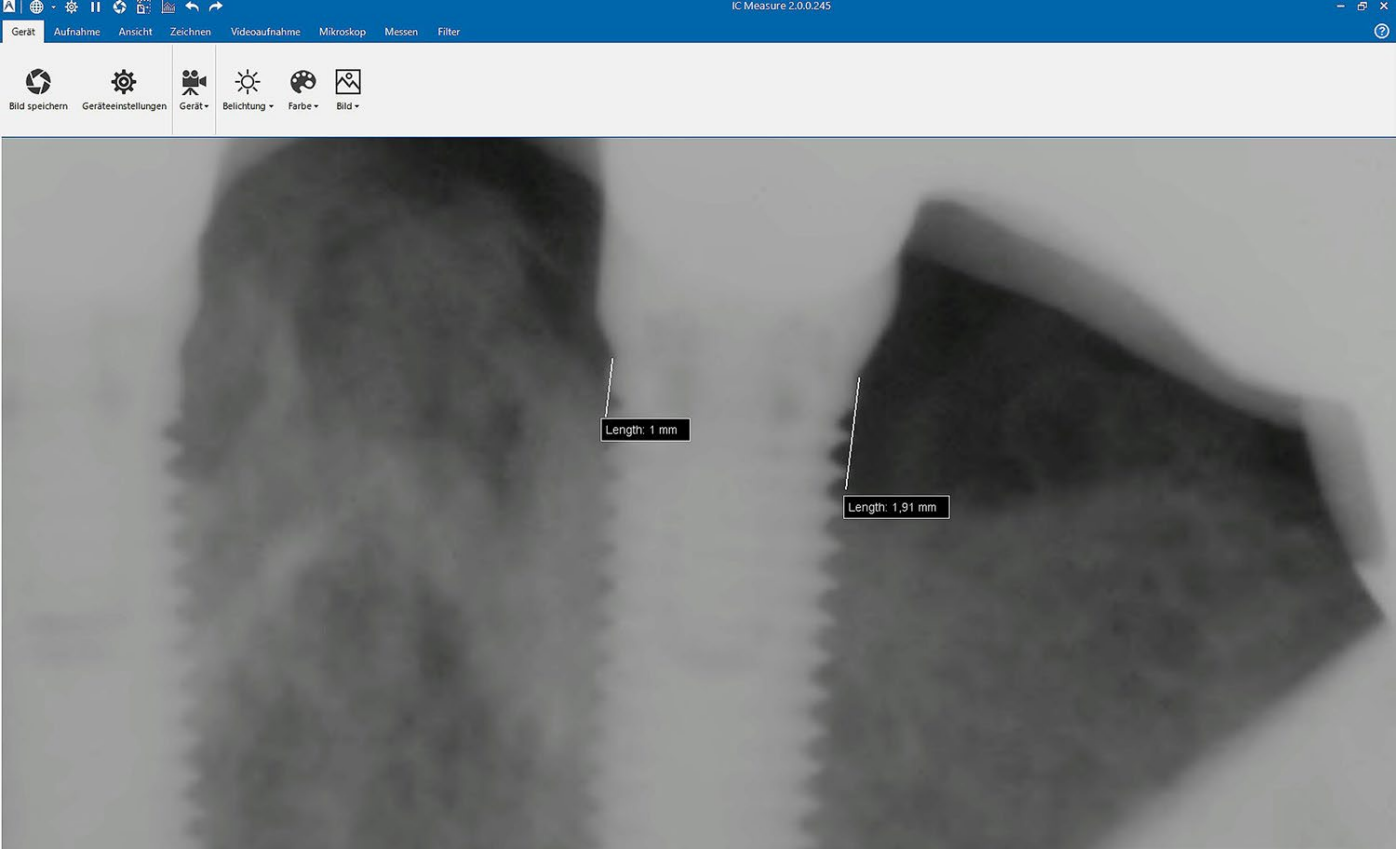
Measurement of the marginal bone loss

Bone resorption was evaluated by measuring the bone crest levels around the implants via standard right-angle parallel technique with single digital radiographs [9, 10] (Fig. 6). The radiographs were scanned at 600 dpi (Trophy RVG UI USB Sensor, KODAK 5.0 software, Carestream, Stuttgart, Germany) and a special image analysis software was used to assess bone level (IC Measure, The Imaging Source Europe GmbH, Bremen, Germany).

PPD was measured in mm at six (mesio-buccal, buccal, disto-buccal, mesio-palatinal, palatinal and disto-palatinal) and BOP was measured at four peri-implant (mesial, buccal, distal and palatinal) sites according to Gerber et al. [11]. The deepest pocket was included in the analysis, and any bleeding on probing was recorded as affirmative.

Plaque accumulation was evaluated using the plaque Index according to Mombelli et al. [12]. Plaque accumulation was evaluated using the plaque Index according to Mombelli et al. [9]. According to that, no detection of plaque was scored as “0”, plaque only recognized by running a probe across the smooth as “1”, plaque can be seen by the naked eye as “2” and abundance of soft matter as “3”, respectively.

**Fig. 7:**
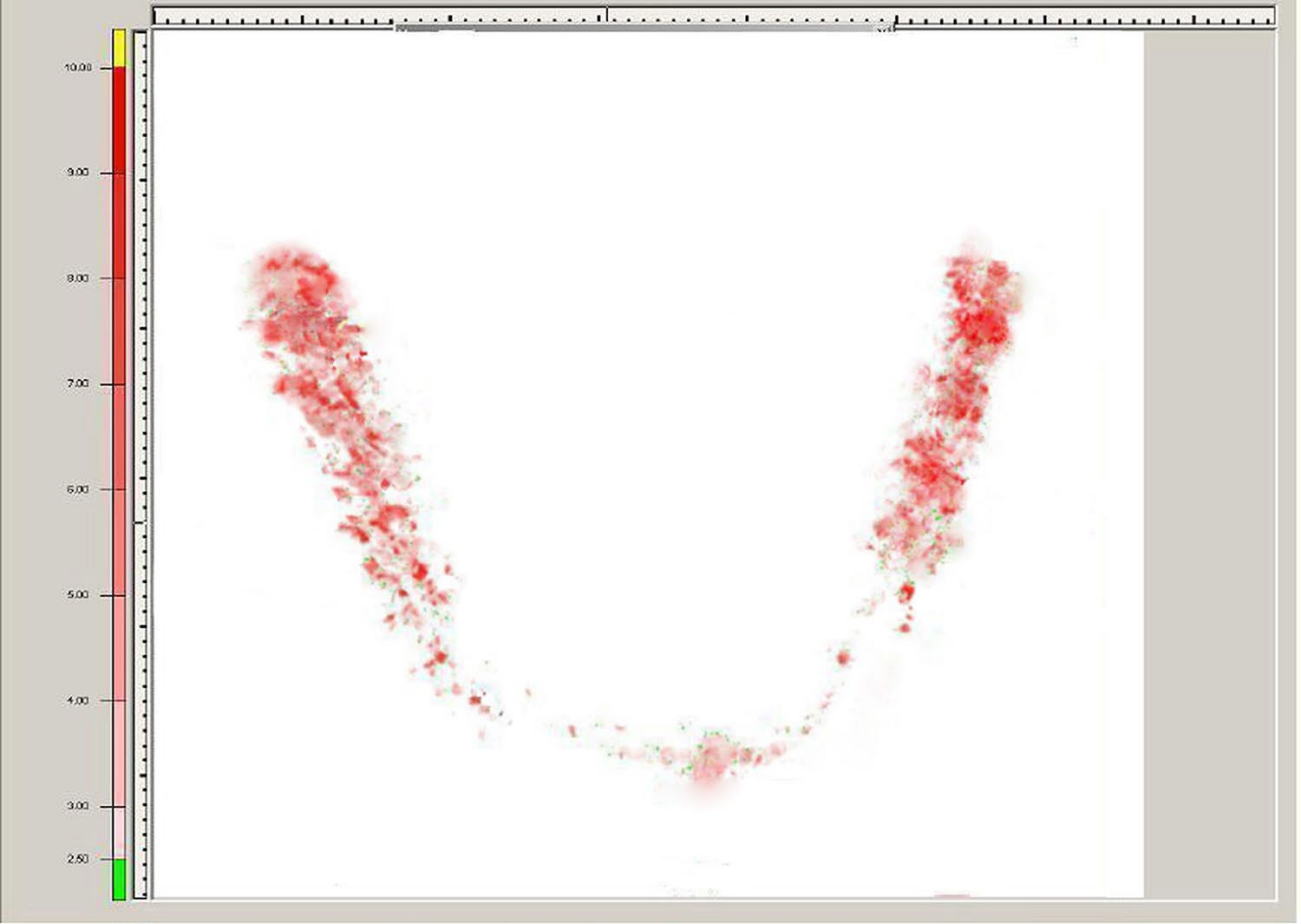
Measurement of occlusal forces using a pressure-sensitive film and its analyzer (Dental Pre-scale 50H type R and Occluzer FPD-703; Fuji Photo Film Co., Japan)

Occlusal forces and their distribution were evaluated using a pressure–sensitive film and its analyzer (Dental Pre-scale 50H type R and Occluzer FPD-703; Fuji Photo Film Co., Japan) as previously described by Ayna et al. [6] (Fig. 7). The measurements were performed before implant insertion with existing dentition/dentures, 1 week after integration of the immediate prosthesis, 1 week after integration of the final prosthetic reconstruction and with 1-year intervals during a period of 6 years.

Patients’ satisfaction and the impact of the reconstruction on the quality of life was assessed by using OHIP11-16, which considers 14 metrics in seven domains using a five-point verbal rating scale ranging from “never” (coded 0) to “very often” (coded 4) [13]. Preoperative OHIP measurement was taken as reference. Low point scores represent a high quality of life.

In addition to the above-mentioned parameters, implant survival rates and prosthetic complications were also documented during the whole observation period.

### Statistical analysis

Study data were analyzed using "Python" (open-source programming language). At first, mean and standard deviation for each group were calculated. The Shapiro–Wilks test was performed to assess the distribution of the parameters. Before statistical evaluation of groups was made, Levene’s test was used to determine homogeneity of variances. The parametric and non-parametric methods were independent *T* test and Mann–Whitney *U* test for group differences and discrete parameters, respectively. The level of significance was set at *p* < 0.05. The Pearson correlation co-efficiency was calculated to analyze the relationship between scale variables.

## Results

The data of 34 patients (17 men and 17 women aged ranged between 52 and 69 (mean: 61 ± 4.59 years) were included. The size of both subgroups was equal (*n*:17). Totally, 136 implants were inserted. All measurements were repeated at yearly intervals.

### Implant loss

During the whole examination period, no implant loss has been observed. In addition, no infectious complications such as fistula formation, sinus empyema or soft tissue inflammation were detected.

### Peak insertion values

The distribution of peak insertion values was shown in Table 1. According to that, similar values were detected and the differences between tilted and straight implants were insignificant.

**Table 1:** Higher values were observed for tilted implants placed in the posterior region (15, 25); however, comparison of peak insertion torque values revealed no significant differences (N/cm)

	15	12	22	25
Acrylic	68.00 ± 5.57	55.70 ± 5.97	56.00 ± 7.36	66.94 ± 6.60
Ceramic	68.29 ± 4.99	53.11 ± 8.44	53.10 ± 7.32	65.88 ± 5.34

### Marginal bone loss

**Fig. 8:**
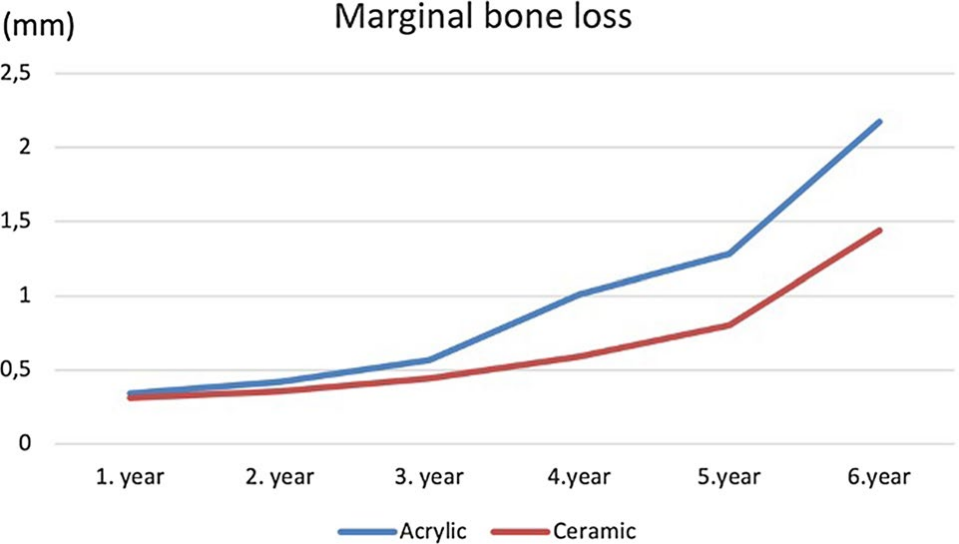
In both groups, a uniform and albeit slight progression of bone loss was observed over the 6-year observation period. Marginal bone loss was significantly more pronounced around the implants in the acrylic group (2.15 ± 0.30) compared to the bone loss around implants of the ceramic group (1.43 mm ± 0.35). Please note the acceleration in both groups from 5 years onward

In both groups, a uniform and albeit slight progression of bone loss was observed over the 5-year observation period. However, an acceleration was detected in both groups after 5 years. The bone loss around implants of the ceramic group remained well within the limits for ‘success’, as defined by the 2007 Pisa consensus over the time (1.43 ± 0.35 mm) [14]. However, marginal bone loss was significantly more pronounced around the implants in the acrylic group (2.15 ± 0.30) and the difference between two groups was statistically significant (*p*: 0.00) There were no significant differences in marginal bone loss between the straight and tilted implants (Fig. 8).

### Plaque accumulation

**Fig. 9:**
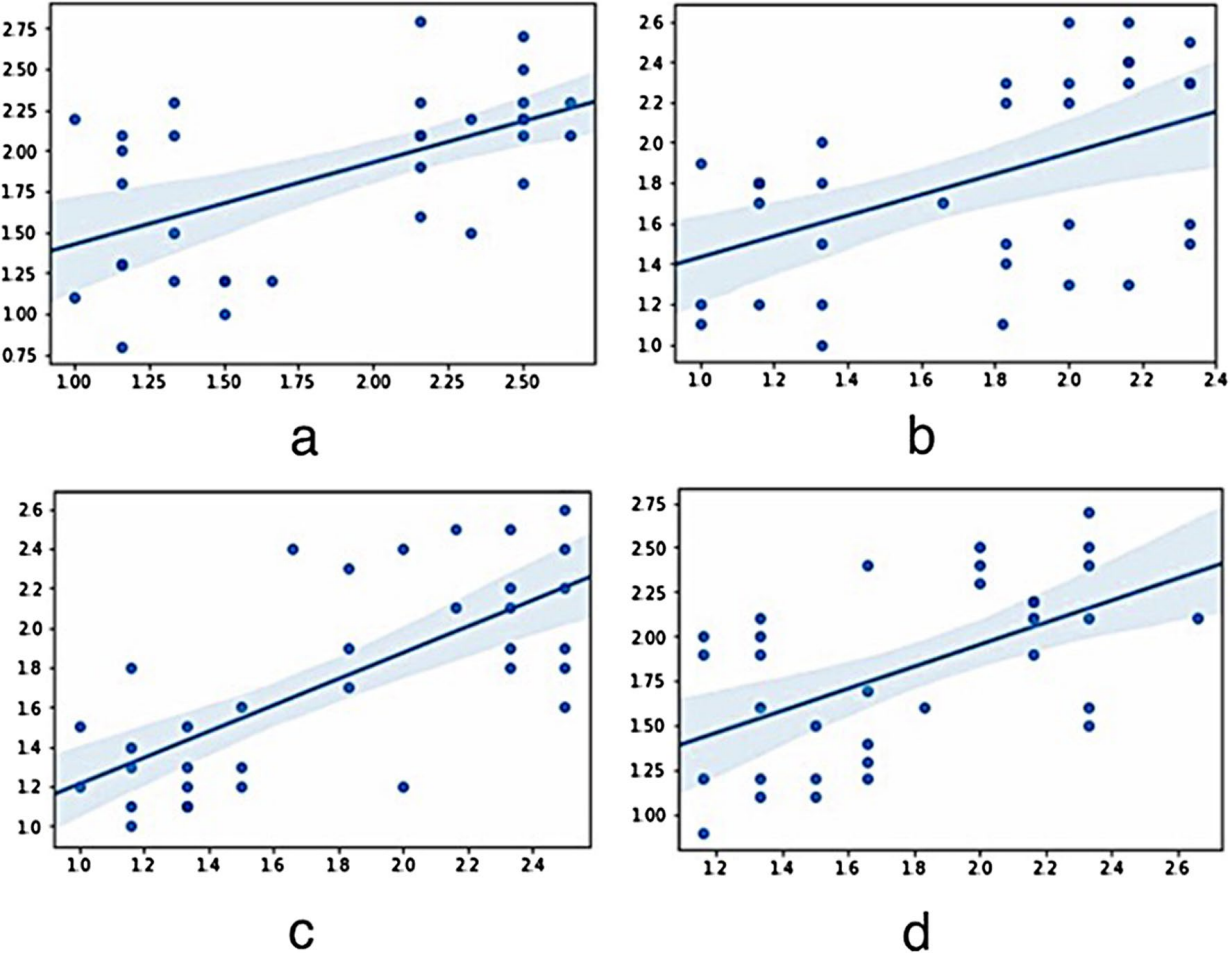
Assessment of the relation between plaque accumulation (0–3) and marginal bone loss showed (mm) a positive correlation in all implant regions. **a** Regio 15 (0.560), **b** Regio 12 (0.492), **c** Regio 22 (0.714) and **d** Regio 25 (0.572) respectively

The plaque index showed that, plaque accumulation was significantly higher in the group with the acrylic superstructures (Table 2). Assessment of the relation between plaque accumulation and marginal bone loss revealed a positive correlation in all implant regions (Fig. 9).

**Table 2:** Comparative analysis of the plaque index values between ceramic and acryl groups regarding the implant regions revealed significantly higher plaque accumulation in the acryl group* (*p*: 0.000)

	15	12	22	25
Acrylic	2.38 ± 0.18*	2.02 ± 0.27*	2.21 ± 0.28*	2.13 ± 0.25*
Ceramic	1.33 ± 0.31	1.42 ± 0.42	1.37 ± 0.37	1.43 ± 0.28

No differences were detected between tilted and straight implants

### Bleeding on probing

**Fig. 10:**
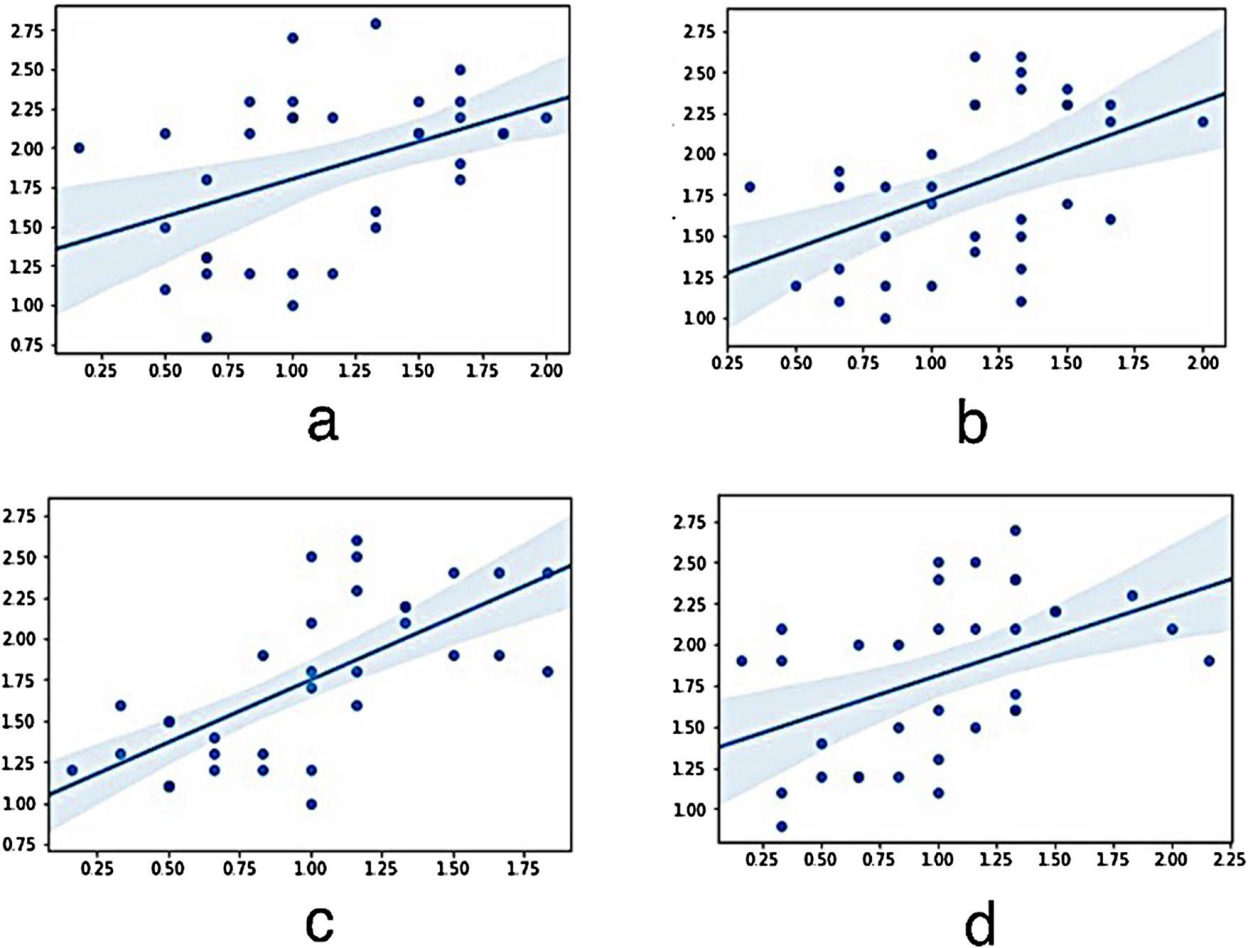
Evaluation of the relation between marginal bone loss (mm) and BOP ( ±) results showed a positive correlation in all implant regions. **a** Regio 15 (0.462), **b** Regio 12 (0.466), **c** Regio 22 (0.681) and **d** Regio 25 (0.434) respectively

Bleeding on probing measurements around the implants revealed no statistically significant differences regarding the implant angulation. However, significantly higher values in the group with acrylic superstructures were observed throughout the examination period over the ceramic superstructure group (Table 3). Evaluation of the relation between marginal bone loss and BOP results showed a positive correlation in all implant regions. (Fig. 10).

**Table 3:** Comparative analysis of the BOP values between ceramic and acryl groups regarding the implant regions revealed significantly higher BOP values in the acryl group* (*p*: 0.000)

	15	12	22	25
Acrylic	1.48 ± 0.30*	1.41 ± 0.24*	1.32 ± 0.30*	1.38 ± 0.33*
Ceramic	0.76 ± 0.28	0.86 ± 0.27	0.65 ± 0.27	0.65 ± 0.29

No differences were detected between tilted and straight implants

### PPD

PPD increased consistently and significantly over time in both groups. Significantly shallower pockets were found at implants supporting the ceramic superstructures (Table 4).

**Table 4:** Comparative analysis of the probing pocket depths (mm) between ceramic and acryl groups regarding the implant regions revealed significant higher values in the acryl group* (*p*: 0.000)

	15	12	22	25
Acrylic	2.71 ± 0.10*	2.61 ± 0.09*	2.63 ± 0.09*	2.73 ± 0.17*
Ceramic	2.02 ± 0.12	1.97 ± 0.07	1.698 ± 0.08	2.01 ± 0.09

No differences were detected between tilted and straight implants

**Fig. 11:**
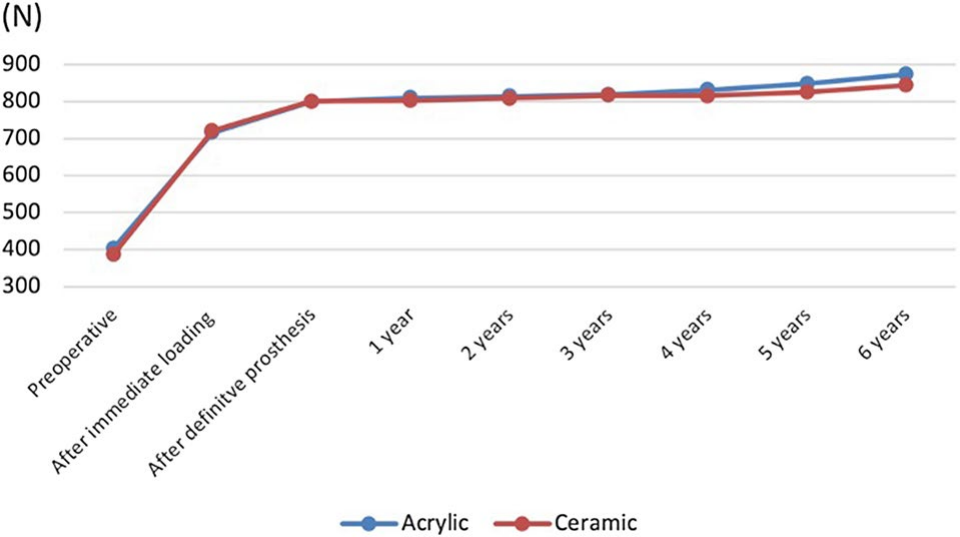
Occlusal force improved immediately after delivering of the immediate prosthesis in both groups. An increasing difference in favor of acrylic superstructures began to evolve from 4 years months onward; however, the difference was statistically not significant. The mean values of deviation regarding the differences in occlusal forces during the whole examination period was 151.41 N for the acrylic and 153.59 N ceramic superstructures, respectively

Occlusal force improved immediately after functional loading in both groups. An increasing difference in favor of acrylic superstructures began to evolve from 4 years months onward; however, the difference was statistically insignificant (Fig. 11).

### OHIP

There was a pronounced subjective improvement, as assessed by the Oral Health Impact Profile (OHIP) score in both groups. There were no differences in the OHIP scores between patients with acrylic dentures and ceramic superstructures (Table 5).

**Table 5:** In both groups, there was a pronounced subjective improvement in OHIP score, which includes 14 items in seven domains (functional limitation, physical pain, psychological discomfort, physical disability, psychological disability, social disability, and handicap) with a five-point verbal rating scale ranging from “never” (coded 0) to “very often” (coded 4)

	Acryl	Ceramic
Preoperative	18.70 ± 2.77	20.76 ± 2.79
After immediate loading	4.35 ± 0.99	4.00 ± 0.86
After definitive prosthesis	3.64 ± 1.05	2.52 ± 0.94
1 year	3.76 ± 1.03	2.94 ± 0.96
2 years	3.76 ± 1.3	3.05 ± 0.89
3 years	3.70 ± 1.1	3.05 ± 1.02
4 years	3.76 ± 1.25	3.17 ± 1.07
5 years	3.82 ± 1.28	3.35 ± 0.93
6 years	7.76 ± 2.53	3.70 ± 0.68

Low point scores represent a high quality of life. No difference has been observed between patients with acrylic dentures and ceramic superstructures

### Prosthetic complications

The survival rates for both immediate and final prostheses were 100%. After adjustment of the immediate provisional prosthesis, dislodgement of the acrylic teeth was observed in five cases. The problem has been resolved by repairing the prosthesis and adjustment of the occlusion in situ.

The same problem has occurred in four cases in the acrylic final prosthesis group after 3, 4, 5 and 6 years. In these cases, the dentures had to be removed and repaired in the laboratory. In addition, loosening of the multi-unit abutment screw was observed in one patient at the implant region 12 (3 years after) and 22 (5 years after) and was resolved by re-tightening the abutment screw.

Detachment of the veneering material was observed by three cases in the ceramic superstructure group after 3, 4 and 5 years following the adjustment. In all cases, dentures were removed and repaired in the laboratory. In the ceramic group, loosening of the multi-unit abutment screw was observed in one patient and was resolved by re-tightening the abutment screw.

All superstructures were easily mended and served well after repair. No further mechanical complications were registered during the observation period. However, abrasion and discoloring of the teeth has been observed nearly by all acrylic superstructures.

## Discussion

In the literature, the number of the studies focusing on the prosthesis related complications in All-on-Four concept is limited. Acrylic prosthesis fracture is reported to be the most common complication followed by provisional screw loosening of the multi-abutment screws for the provisional period [15]. Lopes et al. [16] have stated that, bruxism responsible for a good majority of the fractures of the provisional prostheses. In the current study, no fracture of the prosthesis has been observed.

Fracture of the veneering material is another prosthetic complication, which was also previously reported in several studies. In two studies of Tallarico et al., a prevalence of 7.5% [17] and 10% [18] has been reported. Francetti et al. [19] and Cavalli et al. [20] have described the detachment of the veneer material as the most common complication with a prevalence of 23.2% and 17.7% respectively. In the current study, detachment of the veneering material was observed in 17.6% of the cases and was in accordance with the findings reported by Cavalli et al. In the literature [17, 19], it has been suggested that, most of the problems with veneering materials were observed also in patients with possible parafunctional habits.

Similarly, loosening of the abutment screw is another complication, which was also reported in bruxers [16] more commonly. In the current study, the loosening of the screws has been observed in one case in each group (in the acryl group two times in one patient) with a prevalence of 11.76% and 5.88%, respectively. The problem could be easily resolved in situ, without the need for extra laboratory procedures. However, the early management of the situation is very important, because, due to the inappropriate occlusal forces, fracture of the screws could occur [16].

Prosthetic complications reported herein might differ from the previous studies regarding the lower complication rates [21]. This might be attributed to the fact that, most of the above-mentioned complication could result from bruxism and data of the bruxers were not included in the current study. All superstructure-related problems were well manageable; however, would not have convincingly outweighed the undisputable economic advantage of the acrylic restorations, which was previously mentioned by Ayna et al. [6].

The literature provides strong evidence for preliminary conclusions in favor of ceramic suprastructures, thus acrylic restorations have a higher surface roughness and a greater affinity for biofilm formation, with the consequence of increased exudation in periodontal or peri-implant soft tissues [22, 23]. Therefore, increased bone resorption around implants in the acrylic superstructure group was in accordance with clinical parameters and could be pretty much attributed to the increased inflammatory changes during the whole examination period. However, differences in resilience between both superstructures should be also taken into consideration, thus differences in elastic properties of various superstructures could influence both the occlusal load distribution and marginal bone resorption around dental implants [24].

In the literature, there are number of articles with different follow-up periods, focusing on the marginal bone loss around implants inserted and loaded according to the All-on-Four concept. However, the differences regarding the type of the superstructure have been mostly overlooked. It is well known that, acrylic restorations have a higher surface roughness and a greater affinity for biofilm formation, with the consequence of increased inflammation in periodontal or peri-implant soft tissues [22, 23], which has been also shown to be statistically correlated with the amount of bone resorption around dental implants [25]. The results of the current study revealed an average crestal bone loss for loaded implants in the acrylic group to be 2.30 ± 0.30 mm annually, whereas implants in the ceramic superstructure group showed significantly lower values (1.43 ± 0.35 mm). Besides that, results of the assessment of peri-implantary tissues via BOP and plaque accumulation showed also higher values in the acrylic superstructure group, which corresponds with the results of the marginal bone loss.

In their recent review, Chan and Nuddel [15] have subdivided the studies focusing on assessment of marginal bone loss following All-on-Four concept as short term (< 3 years), medium term (3–7 years), and long term (> 7 years). According to this, marginal bone loss ranges from 0.14 mm ± 0.59 to 1.19 mm ± 0.33, 0.39 mm ± 0.18 to 1.9 mm ± 1.1, and 1.30 mm ± 0.63 SD to 2.30 mm for short term, medium term, and long term, respectively [7, 8, 26]. The results of the present study have also clearly showed the increase in marginal bone loss from 5 years onwards. Considering this, it could be proclaimed that, for the assessment of marginal bone loss, the follow-up period should be at least 5 years to obtain reliable results, because shorter observation periods might lead to different conclusions.

In a comparative analysis of ceramic and bar retained acrylic superstructures according to All-on-Four concept for the edentulous mandible, equivalent levels of marginal bone loss were found over a period of 7 years; however, higher level of plaque accumulation was observed around implants of the acryl superstructure group [27] Recently, Chochlidakis et al. [28] have also reported that, complications such as soft tissue hypertrophy and plaque accumulation were associated with metal-acrylic resin prostheses more often. Apart from the bone loss, the results regarding the plaque accumulation expressed herein were similar to those reported in the literature [27–29]. Therefore, it can be suggested that, patients with acrylic superstructures should be highly motivated to maintain their personal oral hygiene.

The results of the current study showed a significant improvement in bite force immediately after adjustment of the provisional acrylic prosthesis. This finding could have also influenced the OHIP scores, thus masticatory performance is shown to be determinant factor in oral-health-related quality of life [30]. Moreover, slight increase in bite force over the whole examination period could be explained by the adaptation of the masticatory muscles and corresponds to the results of the previous studies [31]. A slight difference between two groups at the 6 years examination could be explained by the fact that, acrylic teeth are subjected more pronounced to extrinsic and intrinsic stains and discolor over time [32].

It is obvious that, bruxism [33], smoking [34], uncontrolled diabetes [35], antiresorptive therapy, corticosteroid therapy [36], etc. could negatively affect the osseointegration process and result in implant failures. In the current study, implant survival rate was determined as 100% and the marginal bone loss was nearly within physiological limits in both groups, which differs from previous studies reporting the middle and long term results of maxillary All-on-Four concept [8, 16, 37, 38]. The highly selected patient group in the present study with the exclusion of risk common risk factors might be viewed as a limitation.

## Conclusion

According to the clinical/radiological results and patient-reported outcomes, both acrylic and ceramic superstructures appeared to be equivalent after 6 years; however, ceramic superstructures revealed superior clinical results in terms of less bone loss and plaque accumulation. The rational choice of superstructures in All-on-Four concept requires comprehensive, long-term observation and careful cost–benefit analysis.
